# Atomic-Level Structural Dynamics of Polyoxoniobates during DMMP Decomposition

**DOI:** 10.1038/s41598-017-00772-x

**Published:** 2017-04-10

**Authors:** Qi Wang, Robert C. Chapleski, Anna M. Plonka, Wesley O. Gordon, Weiwei Guo, Thuy-Duong Nguyen-Phan, Conor H. Sharp, Nebojsa S. Marinkovic, Sanjaya D. Senanayake, John R. Morris, Craig L. Hill, Diego Troya, Anatoly I. Frenkel

**Affiliations:** 1 Department of Material Science and Chemical Engineering, Stony Brook University, Stony Brook, NY 11794 USA; 2 Department of Chemistry, Virginia Tech, Blacksburg, VA 24061 USA; 3 U.S. Army Edgewood Chemical Biological Center APG, MD, 21010 USA; 4 Department of Chemistry, Emory University, Atlanta, GA 30322 USA; 5 Department of Chemistry, Brookhaven National Laboratory, Upton, NY 11973 USA; 6 Department of Chemical Engineering, Columbia University, New York, NY 10027 USA

## Abstract

Ambient pressure *in situ* synchrotron-based spectroscopic techniques have been correlated to illuminate atomic-level details of bond breaking and formation during the hydrolysis of a chemical warfare nerve agent simulant over a polyoxometalate catalyst. Specifically, a Cs_8_[Nb_6_O_19_] polyoxoniobate catalyst has been shown to react readily with dimethyl methylphosphonate (DMMP). The atomic-level transformations of all reactant moieties, the [Nb_6_O_19_]^8−^ polyanion, its Cs^+^ counterions, and the DMMP substrate, were tracked under ambient conditions by a combination of X-ray absorption fine structure spectroscopy, Raman spectroscopy, and X-ray diffraction. Results reveal that the reaction mechanism follows general base (in contrast to specific base) hydrolysis. Together with computational results, the work demonstrates that the ultimate fate of DMMP hydrolysis at the Cs_8_[Nb_6_O_19_] catalyst is strong binding of the (methyl) methylphosphonic acid ((M)MPA) product to the polyanions, which ultimately inhibits catalytic turnover.

## Introduction

Organophosphorus (OP) compounds are typically found in battlefield or agricultural settings and present a health hazard to most living organisms. Highly toxic OP nerve agents such as Sarin, Soman and VX constitute a global risk of growing concern^1–3^, as recently noted in the news. Materials and methods aimed at the sequestration and decomposition of OP compounds have been developed in the past, including oxidation with bleach and related reagents, detoxification by (catalytic) hydrolysis, and degradation involving biochemical approaches^3–5^. However, many of these materials and processes are effective only in the aqueous phase, and thus not practical when OP compounds are deployed as aerosols or gases. Significant efforts have sought to develop effective filtration materials, coatings/fabrics or skin protectants that maintain high efficacy for deactivation of gaseous toxic compounds^6, 7^. Motivated by their noteworthy reactivity, many metal oxide/hydroxide-based formulations^8–11^, metal organic frameworks (MOFs)^12–18^, polyoxometalates (POMs)^19–21^, and relevant composites^22^, have attracted significant recent attention as potential catalysts of nerve-agent decomposition. However, scientists are only beginning to understand the atomic-level details of how chemical warfare agents (CWAs) are transformed on these new catalysts.

Polyoxoniobates (PONbs) have a variable composition, structural polymorphism, and catalytic properties^23, 24^. As such, they are attractive candidates for OP decontamination, as noted in two recent studies that have used polymeric niobates^20, 21^. The experimental work with Cs_8_[Nb_6_O_19_]^21^ has also been recently augmented by a theoretical study of the reaction mechanism at the gas-surface interface involving Sarin^25^. While previous experiments appear to support the prediction that the mechanism follows general base hydrolysis, many atomistic details of the reaction, including the nature of the active sites, their dynamic changes during reaction, and the characterization of intermediates or products, have yet to be experimentally investigated.

This report focuses on characterization of the reaction mechanism between an OP nerve-agent simulant, DMMP^3, 26^ (Figure S1), and an exemplary reactive basic POM, Cs_8_[Nb_6_O_19_], by correlating multiple *in situ* experimental studies with density functional theory (DFT) calculations. By employing a combination of Raman and synchrotron-based X-ray absorption fine structure (XAFS) spectroscopies with X-ray diffraction (XRD), we have been able, for the first time in a POM reaction, to capture structural, electronic, and dynamic changes within the polyanion catalyst unit itself during a reaction: in this case a multi-center hydrolysis. We emphasize that the three types of complementary *in situ* measurements have been performed under nearly identical operational conditions, which enables direct correlation of results across all experiments. Significantly, the studies have been conducted under conditions useful to operational decontamination, and thus the results provide detailed insight into PONb-based hydrolytic decomposition of OP compounds under practical conditions.

Towards the goal of *in situ* and computational studies at the OP-POM interface, we studied the reaction of vaporized DMMP with solid Cs_8_[Nb_6_O_19_]·xH_2_O (naturally hydrated Cs_8_[Nb_6_O_19_], **CsPONb**). In contrast to other [Nb_6_O_19_] alkali salts, the Cs-based analogue exhibits a higher nerve- agent- simulant decontamination rate for reaction on the POM surface than for the analogous reaction in solution^21^, which makes it a promising material for decomposition of OP compounds at the solid-gas interface and greatly broadens the applicability of these materials.

## Results and Discussion

### Density Functional Theory

**Figure 1:**
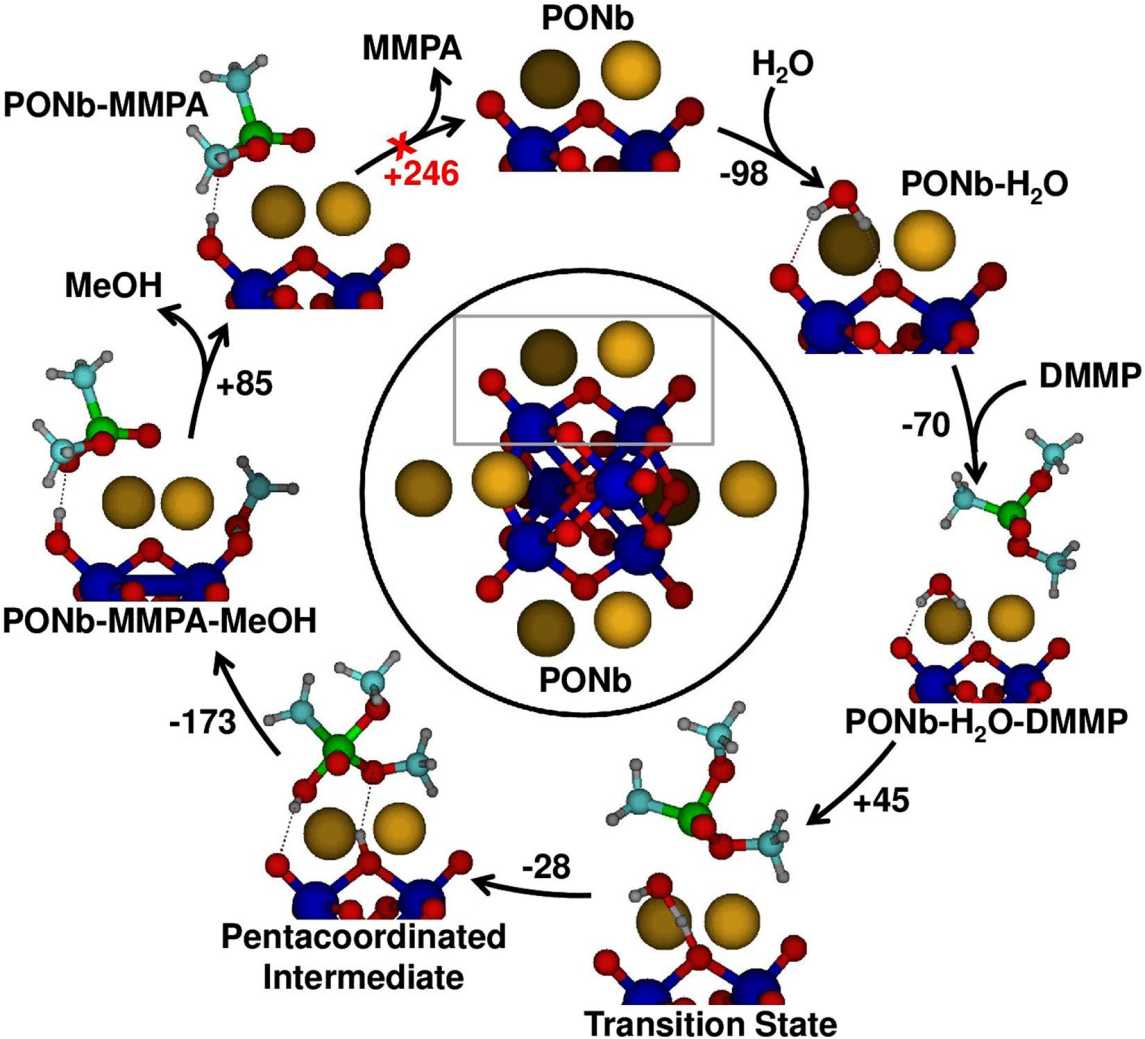
The minimum-energy reaction pathway for the hydrolysis of DMMP by Cs_8_[Nb_6_O_19_]. For clarity, only the region of the PONb unit (outlined by a rectangle) interacting with the adsorbate is shown in the pathway. Energies are in kJ/mol. Color code: red (O), orange (Cs), blue (Nb), grey (H), green (P) and cyan (C).

DFT calculations of the potential energy surface reveal that the overall reaction, DMMP + H_2_O → CH_3_OH + MMPA, (MMPA: methyl methylphosphonic acid) follows a general base hydrolysis mechanism (Fig. 1) where the polyniobate acts as a single site catalyst (optimum geometries of Lindqvist-structure Cs_8_[Nb_6_O_19_] and DMMP are shown in Figure S2). The rate-limiting step of the reaction involves: i) dissociation of a water molecule on the **CsPONb**, which leads to protonation of the polyniobate oxygens and the generation of hydroxide, and ii) nucleophilic addition of the nascent hydroxide to the phosphorus atom of DMMP to generate a pentacoordinated phosphorus intermediate (P5). Both the water dissociation and nucleophilic addition occur concertedly, in a single step. The P5 intermediate undergoes facile dissociation to generate CH_3_OH and MMPA products that remain bound to the **CsPONb**. This complex between products and the **CsPONb** is the most stable stationary point in the reaction pathway, in part due to the strong electrostatic interactions between the electron-rich atoms of the adsorbates and the Cs counterions of the polyniobate. The regeneration of the **CsPONb** requires desorption of both CH_3_OH and MMPA. Notably, the large desorption energy of the phosphonic acid (245.5 kJ/mol) predicts that catalyst reactivation is unlikely under ambient conditions. This lowest-energy pathway with nearly collinear P-OH and P-OCH_3_ bonds at the rate-limiting transition state (two other pathways in which P-OH is collinear with P-CH_3_ or P = O bonds are shown in Figure S3) involves protonation of a bridging oxygen site in the **CsPONb** at the transition state. In order to account for all reaction possibilities and site- specific chemistries, we also analyzed reaction with: i) Cs_8_[Nb_6_O_19_] via protonation at a terminal site, ii) hydrated **CsPONb**, (Cs_8_[Nb_6_O_19_]·14H_2_O), and iii) diprotonated Cs_6_[H_2_Nb_6_O_19_]. Despite slight shifts in the energetics, the reaction mechanism and products do not vary in these systems (Figure S4).

The DFT calculations also suggest important structural and electronic changes within the **CsPONb** during the reaction, which calls for experiments that can capture the dissociation of DMMP and its correlation with atomic changes in the **CsPONb**. A direct comparison of theoretical predictions and results from *in situ* experiments is presented below.

### Raman spectroscopy

**Figure 2:**
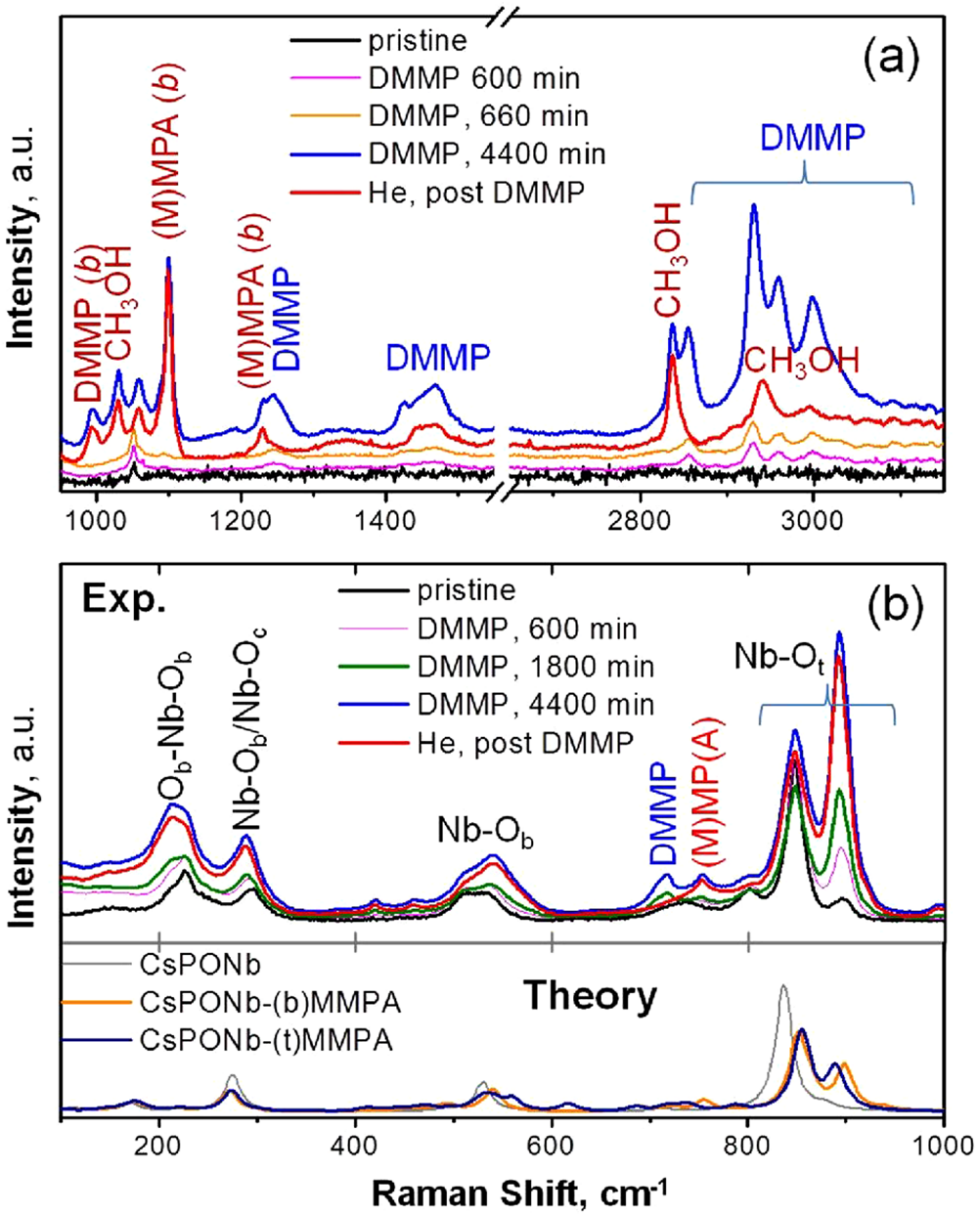
Raman spectra of the reaction system, before reaction (pristine), over the course of stream-feeding of the DMMP/He gas mixture, and in the helium stream following DMMP treatment. (**a**) Spectral region: 950–1550 cm^−1^ and 2650–3150 cm^−1^. (**b**) Spectral region: 100–1000 cm^−1^; Bottom panel illustrates theory-predicted Raman signatures for the Cs_8_[Nb_6_O_19_] (**CsPONb**) and Cs_8_[Nb_6_O_19_] bound to MMPA (**CsPONb**-MMPA) at bridging (b) and terminal (t) sites.

The progressive decomposition of DMMP and concomitant **CsPONb** structural changes were followed with *in situ* Raman spectroscopy, which is very sensitive to molecular modes in oxide surfaces^27, 28^. Raman signatures suggest that at early exposure times, DMMP is the predominant species and adsorbs molecularly onto the **CsPONb** surface^28, 29^. At later reaction times (~660 min), Raman bands emerge corresponding to methanol^30–33^ (2838 cm^−1^/2940 cm^−1^, 1029 cm^−1^) and phosphonic acid^28, 34–38^ (1099 cm^−1^, 1058 cm^−1^, ~753 cm^−1^), adsorbed at the **CsPONb** surface. These bands become dominant as excess DMMP molecules are removed by purging with helium (Table S1, Figs 2a and S5).

The spectral features observed in the 100–1000 cm^−1^ range (Fig. 2b, top panel) are sensitive to the dynamics of metal-oxide bonds^27, 39, 40^, and indicate that the initial PONb sample is partially protonated. Upon DMMP exposure, the Nb-O_terminal_ stretching bands (~846 cm^−1^, 897 cm^−1^) grow initially. The 897 cm^−1^ peak corresponds to the symmetric stretch of the Nb-O_t_, and is an indicator of the presence of CsPONb protonation, because its intensity is enhanced dramatically when any of the oxygen sites becomes protonated. Thus, while in the pristine **CsPONb** the intensity of this band is rather small, the peak grows substantially at reaction times when (M)MPA becomes detectable (~660 min. trace), suggesting that the appearance of the product and the protonation of the polyniobate unit are coupled. The 897 cm^−1^ peak was unchanged upon DMMP removal by helium flushing at ambient conditions, which further supports the high stability of the adsorbed (M)MPA-CsPONb complex. To clarify whether growth of the 897 cm^−1^ band is due to protonation at a terminal or a bridging oxygen atom, we calculated Raman spectra for MMPA products adsorbed on Cs_8_[Nb_6_O_19_] that is protonated at either site (Fig. 2b, bottom panel). Both spectra indicate a significant growth of the higher-energy peak in the Nb-O_t_ region, consistent with the experiment. Taking theory and experiment together, it seems likely that the Cs_8_[Nb_6_O_19_] substrate is protonated at both terminal and bridging sites during DMMP decomposition.

An essential experimental result is that the 897 cm^−1^ band plateaus after prolonged DMMP treatment (Figure S6), pointing toward the inactivation of **CsPONb** upon extended exposure. This result is consistent with the large binding energy of the (M)MPA product to the polyniobate, which likely blocks the active sites of the **CsPONb** toward further reaction with incoming DMMP. The crystal structure and transition between **CsPONb** phases of varying degree of protonation upon DMMP exposure were also assessed *in situ* by using synchrotron powder XRD (Figure S7). The PXRD results suggest a gradual change of long-range order during reaction and agree well with the Raman observations of the perturbative effect of the DMMP decomposition reaction on the **CsPONb** structure and the calculated reaction mechanism. The combined theoretical and experimental investigations indicate that the reaction irreversibly transforms the **CsPONb** sample during the reaction. This finding suggests that **CsPONb** is likely not catalytic in gas-solid reaction conditions without a pathway to regenerate the active sites.

### X-ray absorption fine structure

Synchrotron XAFS was employed in order to further assess element-specific structure and charge properties of the **CsPONb**. Owing its high sensitivity to the electronic structure and local geometry around absorbing elements^41, 42^, the near-edge structure (XANES) was examined first. Upon exposure to DMMP, the strong Cs···O_Lind_ (Lindqvist ion oxygen) interaction weakens as result of PONb protonation^43, 44^, and the Cs^+^ ion becomes more positive relative to the pristine sample, as shown by the enhancement of Cs L_3_-edge white line intensity (Figure S8a). This finding is in agreement with the calculations, as Mulliken population analysis shows an increase in the average charge of Cs^+^ counterions during DMMP hydrolysis, from +0.82e in pristine octahedral Cs_8_[Nb_6_O_19_] to +0.84e in Cs_8_[Nb_6_O_19_]-MMPA. A gradual reduction of the main Nb absorption peaks and concomitant increase in the pre-edge peak (Figure S8b) were also observed, consistent with a change in Nb-O coordination within NbO_6_ octahedra^45, 46^. This finding correlates explicitly with the Raman and DFT results that the conversion of DMMP to (M)MPA is accompanied by protonation of Lindqvist unit [Nb_6_O_19_]^8−^ oxygen sites.

**Figure 3:**
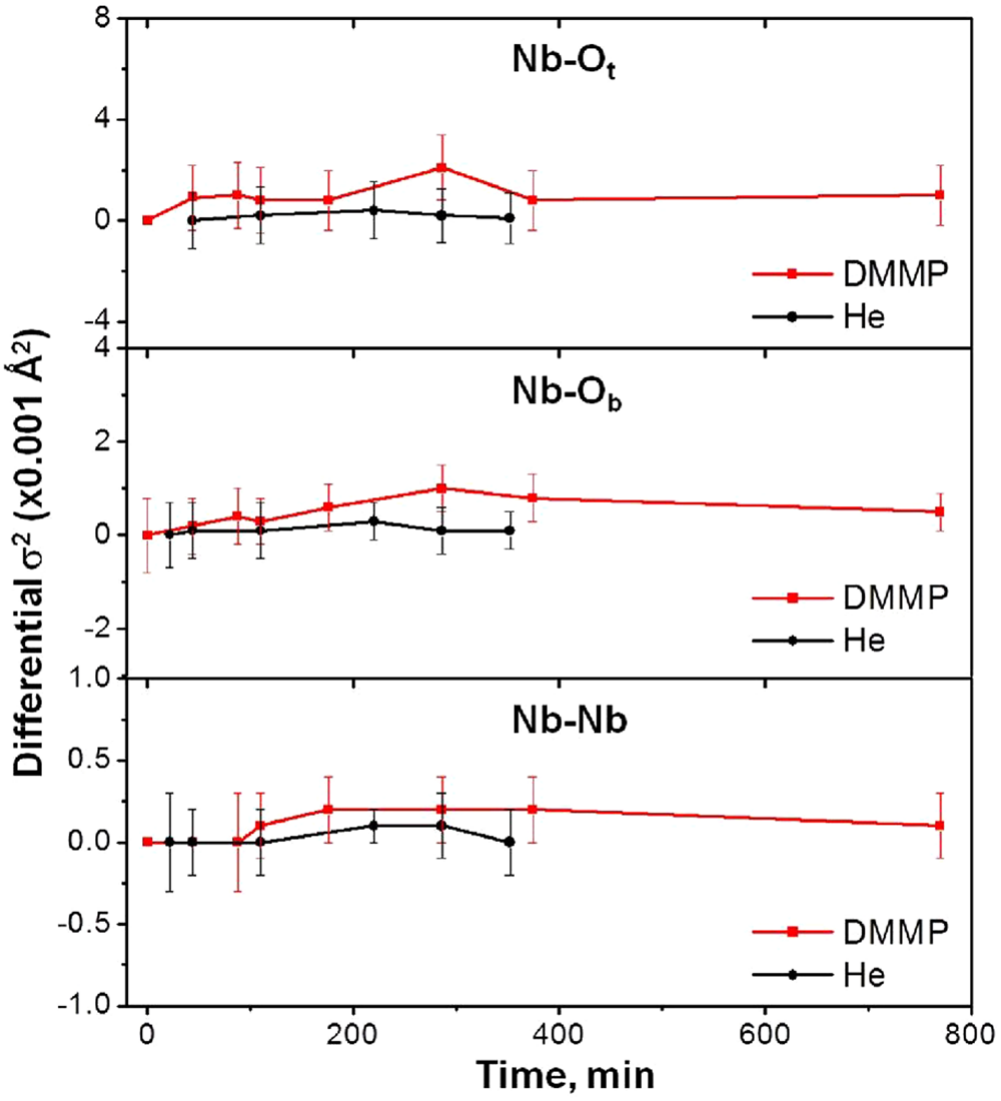
Differential bond distance disorder (σ^2^), as obtained from quantitative analysis of *in situ* Nb K-edge EXAFS data, as a function of DMMP/ He feed during interaction of DMMP with **CsPONb** for (**a**) Nb-O_terminal_, (**b**) Nb-O_bridging_ and (**c**) Nb-Nb bonds in **CsPONb**.

The change in the Nb-O and Nb-Nb bond distance disorders is one of the most important structural descriptors that can bridge experiment and theory. A reduction in amplitudes of Nb K-edge EXAFS peaks for these **CsPONb** bonds was observed upon exposure to DMMP (Figure S9a). Quantitative analysis, using a structure model based on the [Nb_6_O_19_] cluster geometry (Figure S9b), revealed an increase of both Nb-O and Nb-Nb bond distance disorders (σ^2^) during reaction (Figures S9c–f, Table S2). Dynamic changes to **CsPONb** upon continuous exposure to DMMP/He were tracked by *in situ* EXAFS. The disordering of bonds between Nb and nearest-neighbor atoms (Fig. 3) is consistent with the calculations. DFT shows that all Nb-O and Nb-Nb distance disorders increase during reaction (Figure S10), and the larger changes correspond to the oxygen atom that becomes protonated. Thus, at the P5 intermediate of the minimum energy reaction path, the protonation of a bridging oxygen (O_b_) site results in the growth of the Nb-O_b_ bond variance. In products, the O_t_ site is protonated, and the Nb-O_t_ variance is correspondingly large. We note that the duration of the XAFS measurements was much longer than the lifetime of short-lived intermediates in the reaction mechanism; hence, the final states of the reacting complex (**CsPONb**, distorted by the bound products) dominate the bond variances. In addition, the σ^2^ measured in EXAFS consist of both static (${\sigma }_{s}^{2}$) and dynamic $({\sigma }_{d}^{2})$ components that can be considered statistically independent of each other: ${\sigma }^{2}={\sigma }_{s}^{2}+\,{\sigma }_{d}^{2}$ ^47^. In order to establish a direct comparison between experiments and theoretical predictions, we present net changes of bond variances relative to those in the pristine **CsPONb**, averaged over the entire DMMP exposure time. This procedure ensures that the dynamic component, not included in the calculations, will be also omitted from the experimental data.

Table 1 shows that the DFT calculations tend to provide substantially greater σ^2^ values than those derived from the EXAFS experiment. The gap between theory and experiments likely emerges from the different reaction stoichiometries in the measurements and calculations. In the calculations, there is one DMMP molecule per Cs_8_[Nb_6_O_19_] unit, but in the experiment, it is unlikely that each Cs_8_[Nb_6_O_19_] unit is accessible to just a single DMMP molecule. Notwithstanding, the experimental and calculated trends in the variance change can be examined to produce additional insight about the reaction. In the experiment, the increase in the Nb-O_t_ distance disorder is greater than the increase in the Nb-O_b_ disorder. This agrees with the calculations for protonation of the **CsPONb** at an O_terminal_ site. However, the σ^2^(Nb-O_t_)/σ^2^(Nb-O_b_) ratio calculated for that product is significantly larger than in the experiments. The reaction pathway that results in protonation at an O_bridging_ site significantly increases the calculated variance of the Nb-O_b_ distance. These results suggest that a combination of protonation at both terminal and bridging sites may occur under experimental conditions, which is consistent with the Raman study. Regardless of the protonation site, we can conclude that the observed DMMP decomposition and **CsPONb** structural changes in our experiments are mainly associated with the reaction that involves the protonation of the original neutral **CsPONb**. This protonation process emerges as the key step in the overall mechanism and a critical component of the atomic structural response to the reaction when **CsPONb** hydrolytically decomposes DMMP to (M)MPA and methanol. This finding provides new insight into the structure-activity of PONbs that must be considered in future design of polyoxometalates for decomposition of OP compounds and for hydrolysis processes in general.

**Table 1:** Bond Disorder (σ^2^, in Å^2^): Theory vs. Experiment.

Bond Type	Theory	Theory	Experiment
	(O_t_-H^+^)	(O_b_-H^+^)	
Nb-O_t_	0.00605	0.00004	0.00106 (18)
Nb-O_b_	0.00071	0.00274	0.00054 (13)
Nb-Nb	0.00082	0.00401	0.00011 (5)

## Conclusions

In summary, we used a combination of *in situ* techniques (Raman, XAFS, XRD) and associated DFT calculations to reveal detailed structural changes including subtle variations to bond distances in a polyanion unit itself during a POM reaction. In this initial case, the polyniobate, Cs_8_[Nb_6_O_19_] (**CsPONb**) and the much studied organophosphorus hydrolysis substrate, DMMP, were chosen for study. To our knowledge, this is the first time that a structure-activity correlation in POM chemistry has been characterized *in situ*. The results reveal a direct connection between DMMP decomposition and **CsPONb** protonation that induces bond distance disordering and charge redistribution in the catalyst during reaction. The study indicates that the OP compound hydrolytically decomposes via the cleavage of a P-OR bond and affords data of value to application of such POMs in protective filtration materials for OP agents at the gas-surface interface. The fundamental insights into the specific role of niobate sites in the degradation of nerve-agent simulants will be important for the design of new POMs with improved properties for decontamination. In particular, the process of protonation, coupled with the irreversible binding of products, strongly suggests that the **CsPONb**-based materials for OP catalysis may not be viable without new approaches that address product inhibition.

## Methods

### Filtration activity

The Cs_8_[Nb_6_O_19_] sample was prepared and purity assessed following the procedure by Nyman and co-workers^44^. Solid state Cs_8_[Nb_6_O_19_]·xH_2_O (**CsPONb**) was investigated *in situ* for the decomposition of dimethyl methylphosphonate (DMMP) by combined use of Raman spectroscopy and a quadrupole mass spectrometer (QMS, Stanford Research Systems). To simulate the spreading of real agents in the field, a continuous vapor stream of DMMP was generated with a saturator cell developed at Edgewood Chemical and Biological Center^48^. High-purity helium carrier gas, at a flow rate of 10 mL/min, was passed through a glass saturator filled with liquid DMMP. The saturator was suspended in a bath at a constant temperature of 40 °C, in order to properly generate saturated vapor. The measurements were performed by employing a reactor, which consists of a quartz capillary (2.4 mm ID) connected with gas lines by Swagelok style. The **CsPONb** was loosely packed inside the capillary. The sample bed was nicely fixed in position by quartz wool at both ends and then aligned to the focus spot of a Raman optical probe. The DMMP/He mixture from the saturator was stream fed at constant flow rate to the capillary reactor. The outlet of the reactor was connected to the QMS for analysis of flowing mixtures. Such a setup allows a stream feed of DMMP/He gas mixture over the **CsPONb** sample during the acquisition of Raman data. The chemical fragments/ions of mass-to-charge (m/z) = 124 ([C_3_H_9_O_3_P]^+^·), 94 ([C_2_H_7_O_2_P]^+^·), 79 ([CH_4_O_2_P]^+^) and 47 ([PO]^+^) were readily detected by in-line mass spectrometry of the reactor effluent, confirming the steady vaporization and supply of DMMP in the gas stream. The decomposed products were monitored via Raman spectroscopy.

### Raman spectroscopy

Raman spectra were acquired using a Bay Spec spectrometer equipped with 532 nm laser excitation. A non-contact fiber optic probe was used to focus the beam on the sample and collect scattered signals. The data were recorded in continuous mode at a rate of 30 minutes per spectrum, concurrent with DMMP/He stream feed to a **CsPONb**-packed capillary cell. Vibrational bands were identified and assigned through reference to literature and comparison with DFT calculated spectra.

### XAFS experiments

XAFS experiments were performed at Stanford Synchrotron Radiation Lightsource (SSRL), Beamline 2–2 and Advanced Photon Source, Beamline 9BM. Nb K-edge (18986 eV) and Cs L_3_-edge (5012 eV) data were collected in transmission and fluorescence modes, respectively. Ti K-edge (4966 eV) was used for energy calibration in the Cs L_3_–edge experiment. For *ex situ* tests, **CsPONb** sample powder was placed in a sealed jar (~120 mL) that contained a trace amount of liquid DMMP (~50 μl) for a desired interval of time, and the powder sample uniformly brushed on a tape was measured. *In situ* filtration and effluent detection were done by following a procedure similar to that in the Raman experiment. **CsPONb** powder was packed in a Clausen reactor with a Kapton capillary (1.0 mm ID) and the sample bed was aligned to x-ray beam for the proper measurement. Sequential XAFS data were collected in continuous mode with ~20 minutes per spectrum. XAFS data were processed and analyzed using the IFEFFIT package^49, 50^. Quantitative analysis of Nb K-edge EXAFS was performed by fitting theoretical EXAFS spectra to the experimental data in r-space.

### *In situ* XRD experiments

*In situ* powder XRD (PXRD) measurements were conducted during the filtration process at the Advanced Photon Source (APS), Beamline 17BM. The time-resolved PXRD data were collected at 1 minute intervals using the beam of λ = 0.72959 Å and a Perkin-Elmer 2D image detector.

### Computational Analysis

All theoretical calculations were performed using the Gaussian 09 suite of software^51^ with the M06L functional. Geometries and harmonic frequencies of reagents, products, and stationary points along the reaction pathway were optimized using the 6–31 G(d,p) basis set for nonmetal atoms and the Lanl2dz basis set and pseudopotentials for metal atoms. Energies were refined using single-point calculations with the 6–31 +  + G(d,p) basis set for nonmetal atoms and corrected by the zero point. Raman activities were calculated at the M06L/(6–31 +  + G(d,p)—Lanl2dz) level and compared directly with experiment. An ultrafine integration grid was employed in all calculations except for Raman activities, for which a superfine integration grid was used.

## Electronic supplementary material

Supplementary Information
