# Supplementary material for: Atomic-Level Structural Dynamics of Polyoxoniobates during DMMP Decomposition
Source: Sci Rep. 2017 Apr 10;7:773. doi: 10.1038/s41598-017-00772-x (PMC5429595; doi:10.1038/s41598-017-00772-x)
Supplement: Supplementary file 1 — Supplementary Information [file 41598_2017_772_MOESM1_ESM.pdf]

Atomic-Level Structural Dynamics of Polyoxoniobates during DMMP Decomposition

Qi Wang,[1] Robert C. Chapleski Jr.,[2] Anna M. Plonka,[1] Wesley O. Gordon,[3] Weiwei Guo,[4] Thuy-Duong Nguyen-Phan,[5] Conor H. Sharp,[2] Nebojsa S. Marinkovic,[6] Sanjaya D. Senanayake,[5] John R. Morris,[2] Craig L. Hill, Diego Troya,[4] and Anatoly I. Frenkel[1,*]

[1]Department of Material Science and Chemical Engineering, Stony Brook University, Stony Brook, NY 11794, USA

[2]Department of Chemistry, Virginia Tech, Blacksburg, VA 24061, USA

[3]U.S. Army Edgewood Chemical Biological Center APG, MD 21010, USA

[4]Department of Chemistry, Cherry L. Emerson Center for Scientific Computation, Emory University, Atlanta, GA 30322, USA

[5]Department of Chemistry, Brookhaven National Laboratory, Upton, NY 11973, USA

[6]Department of Chemical Engineering, Columbia University, New York, NY 10027, USA

*Correspondence to: anatoly.frenkel@stonybrook.edu

Supporting Information

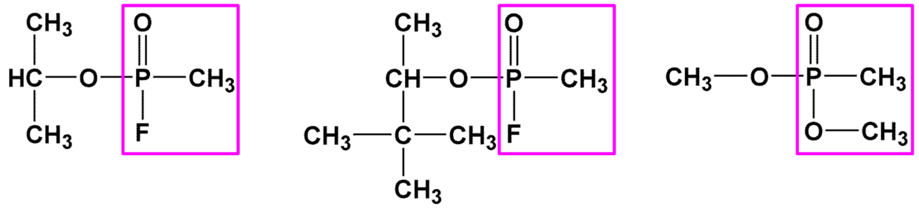

Sarin Soman DMMP

Figure S1: Structures of the G-type chemical warfare agents Sarin (GB) and Soman (GD), and the simulant dimethyl methylphosphonate (DMMP). The highlights illustrate chemical resemblance of DMMP with GB/GD.

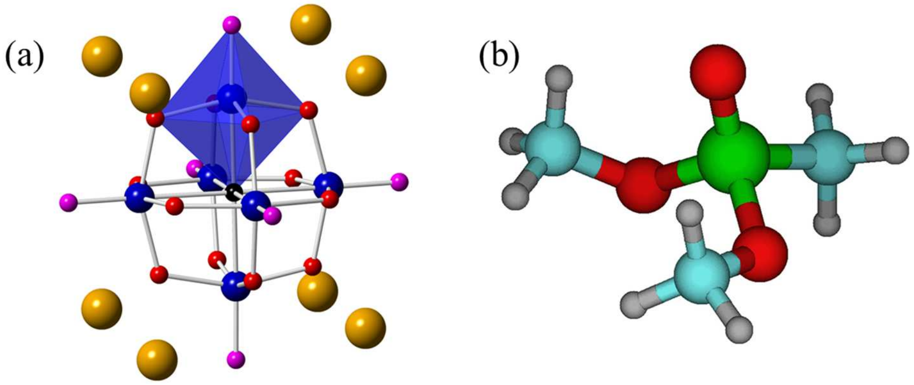

Figure S2: Ball-and-stick representation of (a) $Cs_8[Nb_6O_{19}]$ and (b) dimethyl methylphosphonate (DMMP). The highlighted octahedron (blue) represents the structural unit of the PONb - NbO_6. Color code: Cs: orange, Nb: blue, P: green, C: cyan, H: gray, bridging O: red. Special note: to illustrate the different oxygen sites in $[Nb_6O_{19}]^{8-}$, black and magenta colors are used for central O (O_c) and terminal O (O_t), respectively.

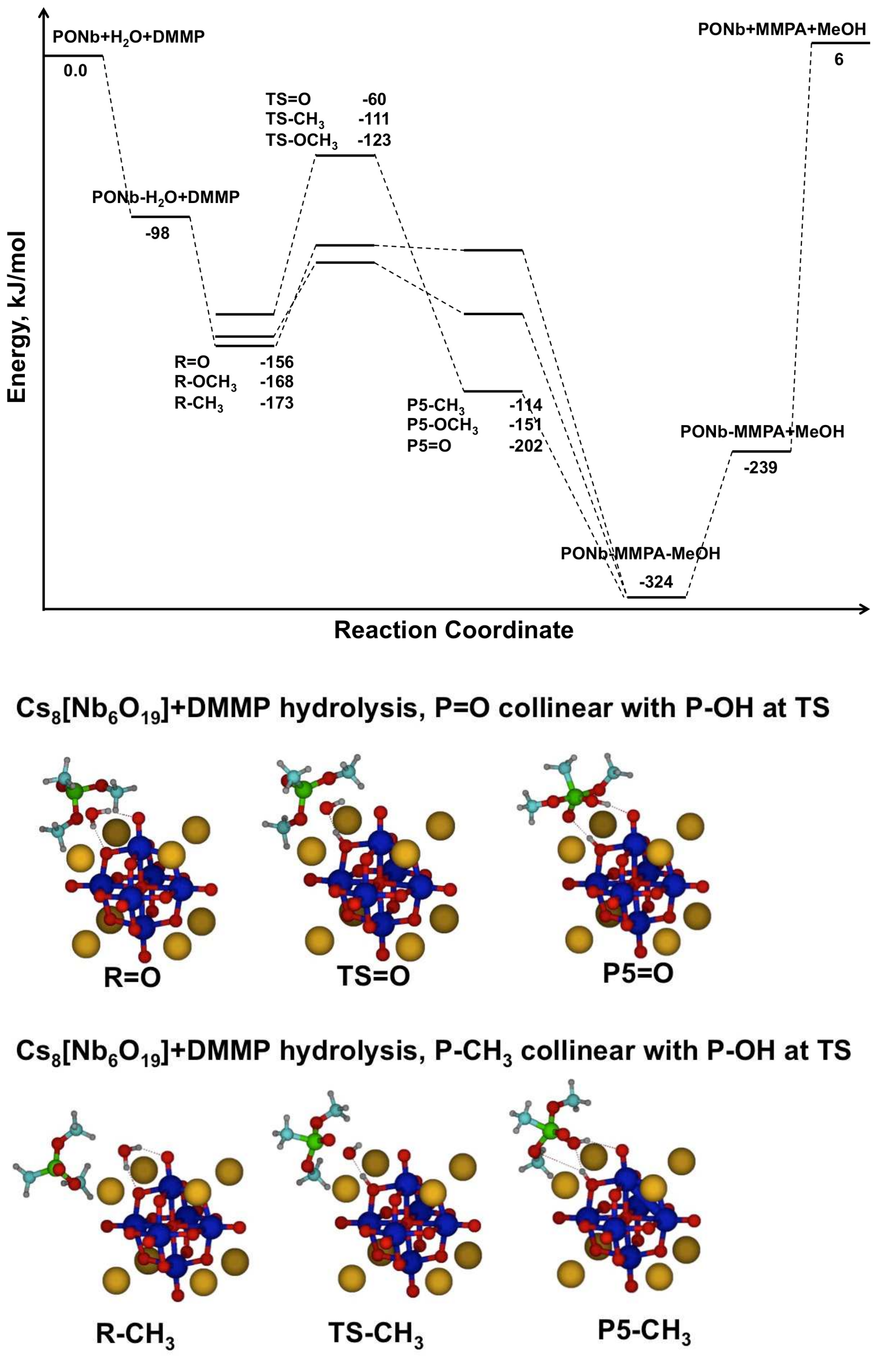

Figure S3. Theory-calculated minimum-energy reaction pathways for the hydrolysis of DMMP with $Cs_8[Nb_6O_{19}]$. Pathways differ by the orientation of DMMP. Pathways in which the P=O and P-CH_3 bonds are in an axial position along with the forming P-OH bond at the transition state are compared to the lowest-barrier pathway (45 kJ/mol, 62 kJ/mol, 96 kJ/mol in barriers, respectively, for P-OCH_3, P-CH_3 and P=O in axial positions, Figure 1). Optimized geometries of each stationary point are given below the reaction pathway. For structures, we used the same color schemes as in Figure 1.

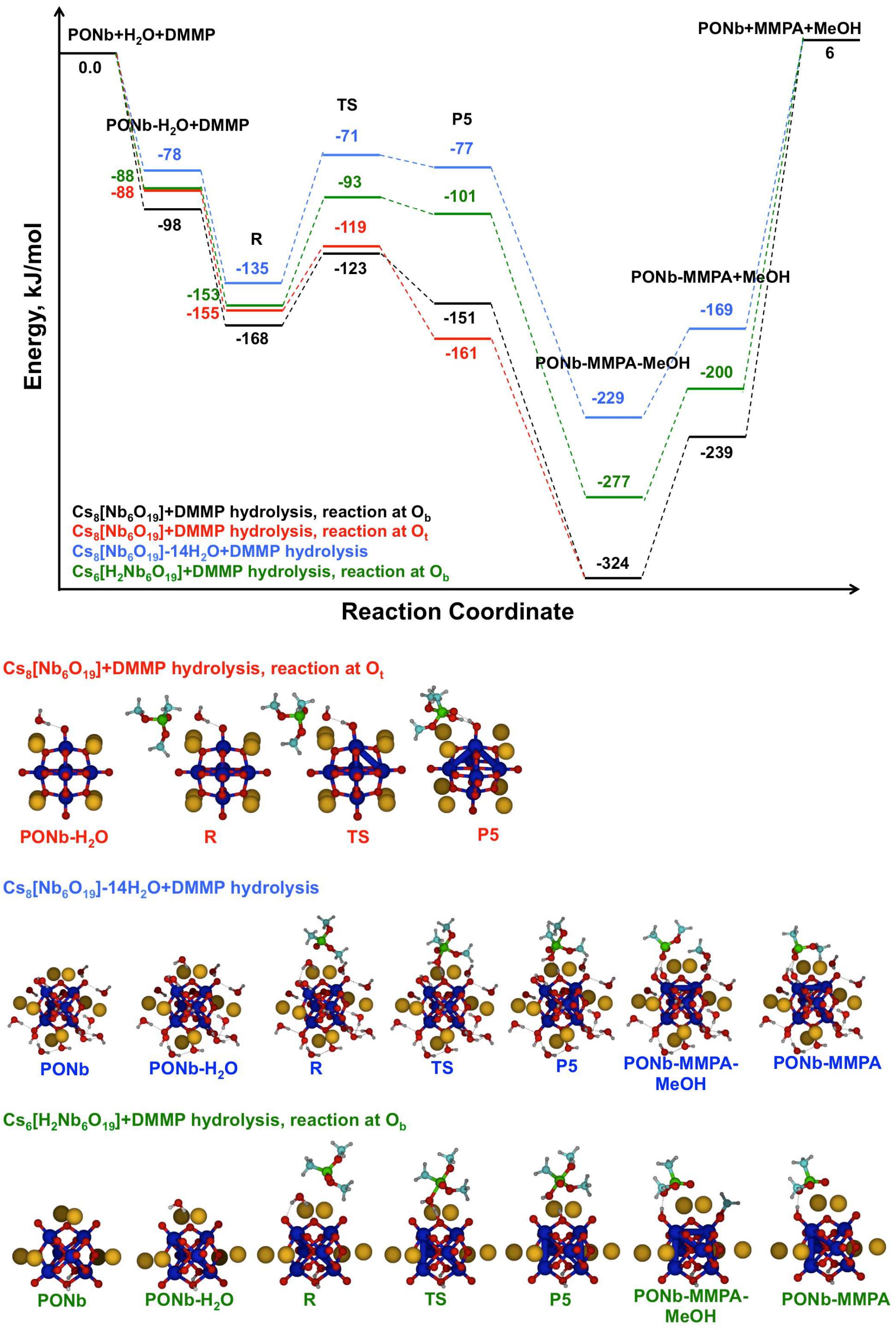

Figure S4. Theory-calculated minimum-energy reaction pathways for the hydrolysis of DMMP with different configurations of **CsPONb**: (i) $Cs_8[Nb_6O_{19}]$, protonated at $O_{terminal}$ site (red). (ii) $Cs_8[Nb_6O_{19}]\cdot 14H_2O$ (blue); a superfine integration grid was implemented in the optimization and single-point energy calculations of points along this hydrated pathway. (iii) $Cs_6[H_2Nb_6O_{19}]$ (green); compared with that of DMMP hydrolysis with $Cs_8[Nb_6O_{19}]$ and protonated at $O_{bridging}$ site (black). Optimized geometries of each stationary point are given below the reaction pathway. For structures, we used the same color scheme as in Figure 1.

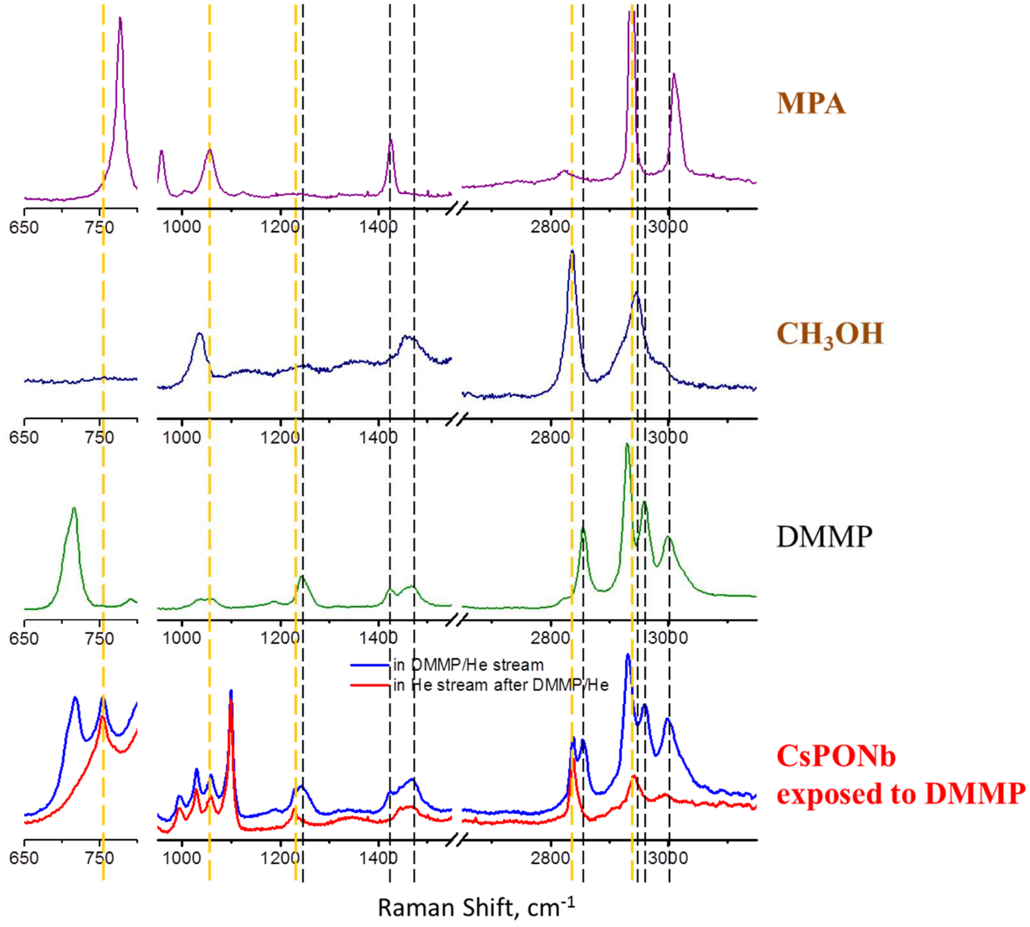

Figure S5: Raman spectra (650-800 cm^{-1}, 950-1550 cm^{-1}, 2650-3150 cm^{-1}) for **CsPONb** treated by DMMP/He stream and further by Helium, compared against with the spectra of the reference compounds DMMP, CH_3OH, methylphosphonic acid (MPA). Dashed lines in black: Raman signatures for the reactant; Dash lines in orange: Raman signatures for the products.

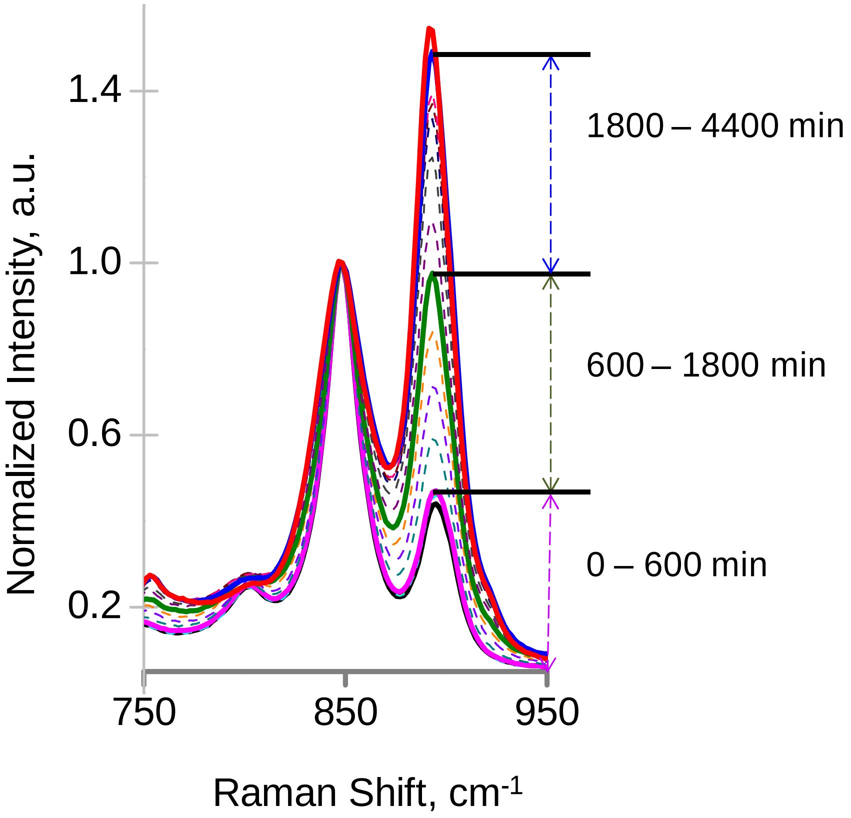

Figure S6: Raman spectra (750-950 cm^{-1}) of intensity normalized to unity at 848.52 cm^{-1} for **CsPONb** by DMMP/He stream. The change of 897 cm^{-1} peak, in various reaction stages, is evident.

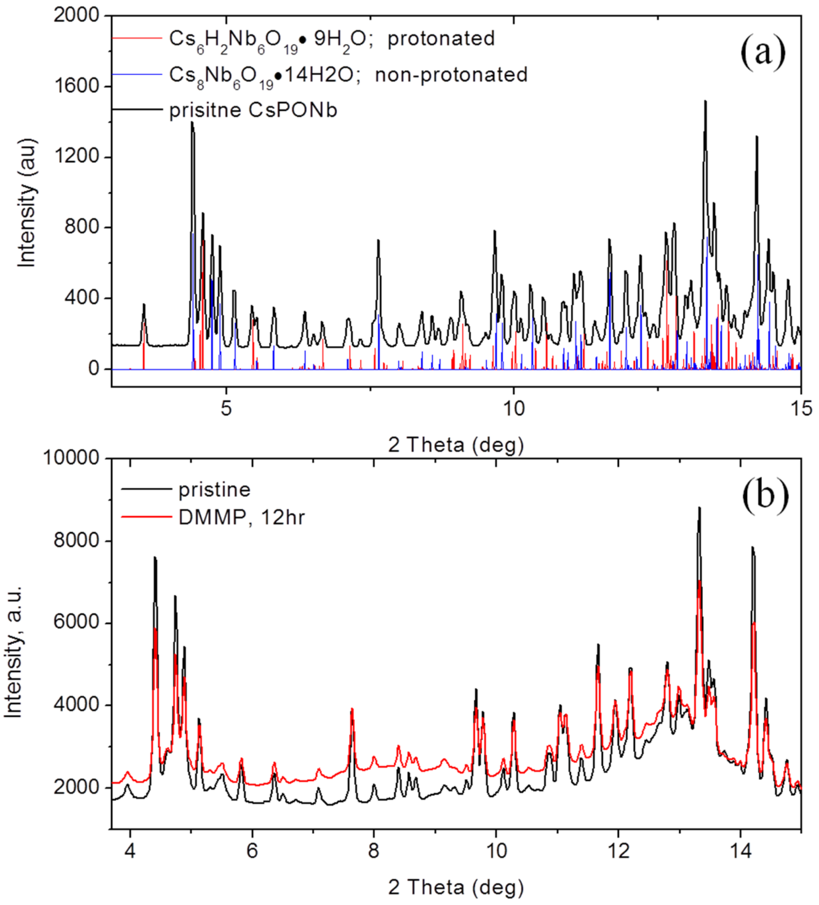

Figure S7: (a) X-ray powder diffraction (XRD) pattern for pristine **CsPONb**, plotted against reference patterns for two major phases of **CsPONb**: protonated ($Cs_6[H_2Nb_6O_{19}]\cdot 9H_2O$) and nonprotonated ($Cs_8[Nb_6O_{19}]\cdot 14H_2O$); (b) XRD pattern for **CsPONb** before (black) and after stream-fed DMMP/He for 12hr (red).

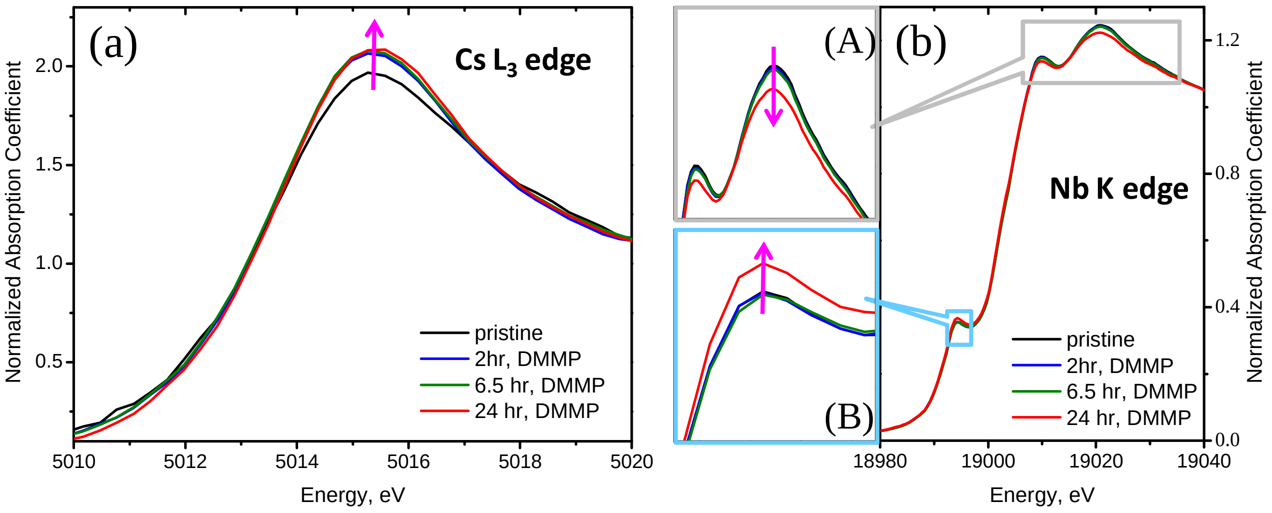

Figure S8: (a) Cs L_3-edge and (b) Nb K-edge XANES spectra for **CsPONb** before and after exposure to DMMP vapor for 2hr, 6.5hr and 24 hr. The insets of Fig (b) illustrate the change of the Nb K-edge white line (top) and pre-edge features (bottom).

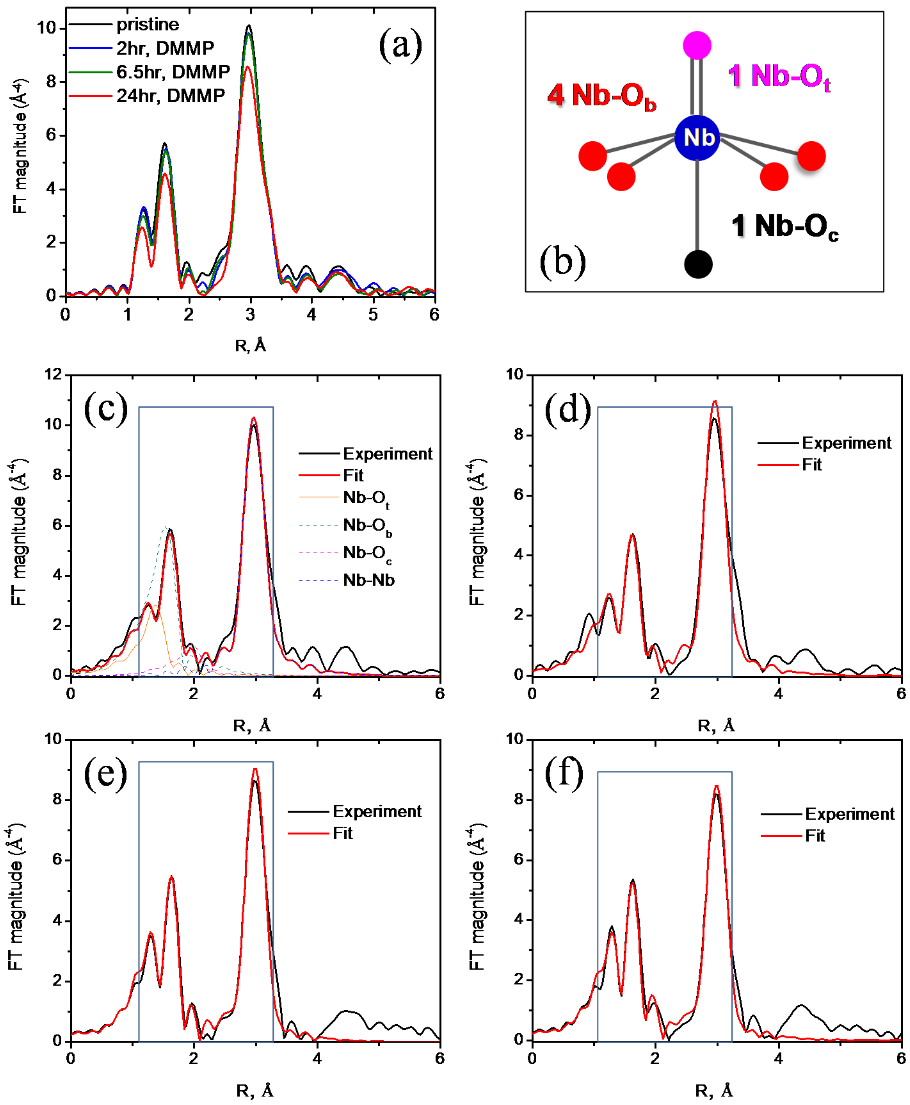

Figure S9: (Top) Nb K-edge EXAFS data and theoretical fits: (a) Fourier-transform magnitudes of the Nb K-edge EXAFS spectra for **CsPONb** before and after exposure to DMMP vapor for 2hr, 6.5hr and 24 hr; (b) Illustration of NbO_6 octahedron in a Lindqvist cluster. A structural model for Nb EXAFS fit was constructed by including three types of Nb-O bonds and the nearest Nb-Nb bond, with coordination numbers set at 1, 4, 1, and 4 for the Nb-O_t, Nb-O_b, Nb-O_c, and Nb-Nb bonds.

(Bottom) Fourier transform magnitudes for the FEFF6 theory fit (red) to the experimental data (black) at Nb K-edge for **CsPONb**: (a) powder sample before DMMP exposure; (b) powder sample after exposure to DMMP vapor for 24hr; (c) ~ 0.03 M solution sample; (d) ~ 0.03 M solution sample after mixed with ~ 0.03 M DMMP for 47 hr.

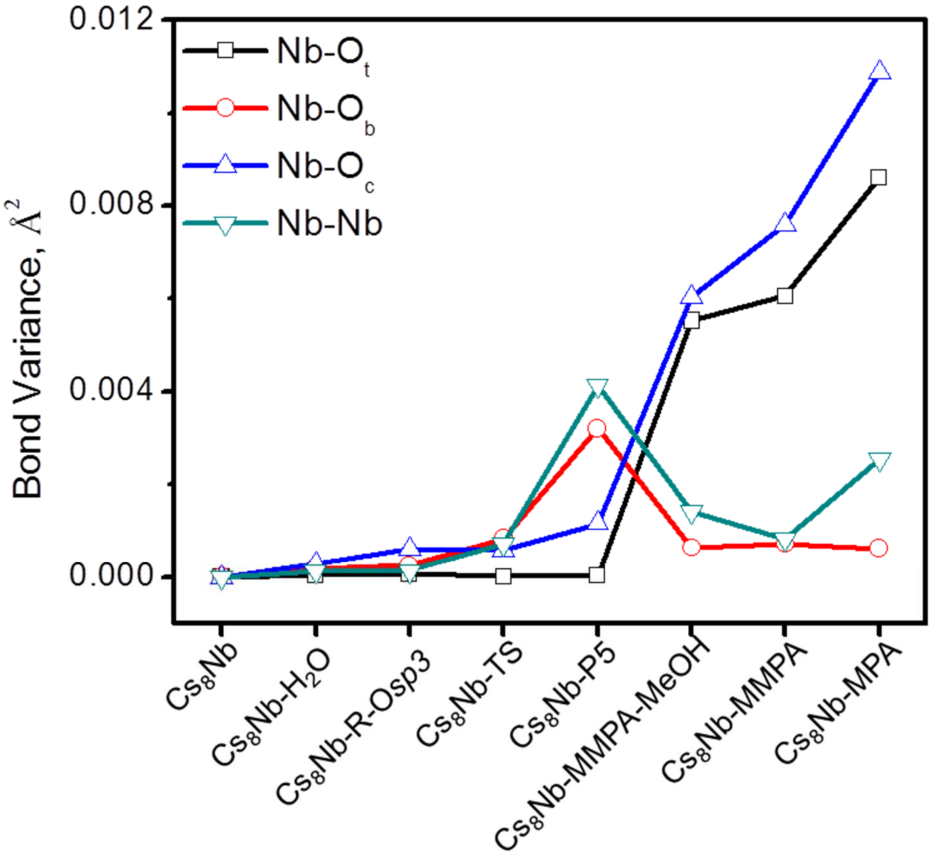

Figure S10: Theory-derived bond distance variances for selected states along the lowest energy reaction pathway of DMMP hydrolysis with $Cs_8[Nb_6O_{19}]$.

Table S1. Raman Spectral Assignments in Conversion of DMMP to (M)MPA

DMMP Peak Position (cm^{-1})	DMMP Peak assignment [1,2]	Products Peak Position (cm^{-1})	Products Peak assignment
2999	ν_a((P)CH_3)		
2959	ν_s((O)CH_3)	2940	ν_a(CH_3), CH_3OH (*f*) [3-6]
2930	ν_s((P)CH_3)		
2855	ν_s((O)CH_3)	2838	ν_s(CH_3), CH_3OH (*f*) [3-6]
1467	δ_a((O)CH_3)		
1423	δ_s((O)CH_3)		
1240	ν(P-O)	1228	ν_a(O-P-O), (M)MPA (*b*)
		1099	ν_s(O-P-O), MMPA (*b*)
1059	ν(C-O(P)), ν(O-P-O)	1058	ν (C-O(P))[6] , ν (PO_3) [7,8]
1032		1029	ν (C-O), CH_3OH (*f*) [4,5]
		993	DMMP (*b*) (from DFT)
717	ν (P-C)	753	ν (P-C), MPA and anion, [7-10] MMPA (*b*)[1]

Note: 1) DMMP: dimethyl methylphosphonate,
MMPA: methyl methylphosphonic acid,
MPA: methylphosphonic acid.
2) (*b)*: bound or absorbed species; (*f)*: unbound species

Table S2: Best fit results for the structural parameters obtained from the **CsPONb** EXAFS data analysis

Structure Parameters	$Cs_8[Nb_6O_{19}]\cdot 14H_2O$			**CsPONb**-DMMP Gas-Phase Reaction			**CsPONb**-DMMP Solution Reaction	
	XRD* [11]	Theory		Before DMMP	DMMP, 24hr		Before DMMP	DMMP, 47hr
$R_{Nb\text{-}Ot}$ (Å)	1.797	1.8323		1.77 (2)	1.77 (1)		1.775 (6)	1.770 (6)
$R_{Nb\text{-}Ob}$ (Å)	1.994**	2.0173		1.97 (1)	1.97 (1)		1.968 (5)	1.967 (6)
$R_{Nb\text{-}Oc}$ (Å)	2.385	2.3922		2.38 (3)	2.41 (5)		2.42 (3)	2.44 (3)
$R_{Nb\text{-}Nb}$ (Å)	3.346**	3.3831		3.326 (6)	3.322 (7)		3.335 (3)	3.335 (3)
$\sigma^2_{Nb\text{-}Ot}$ (Å^2)				0.0029 (14)	0.0023 (10)		0.0015 (4)	0.0014 (4)
$\sigma^2_{Nb\text{-}Ob}$ (Å^2)				0.0053 (12)	0.0068 (9)		0.0060 (5)	0.0066 (6)
$\sigma^2_{Nb\text{-}Oc}$ (Å^2)				0.0032 (28)	0.0076 (65)		0.0072 (38)	0.0058 (29)
$\sigma^2_{Nb\text{-}Nb}$ (Å^2)				0.0039 (6)	0.0044 (2)		0.0045 (2)	0.0047 (2)

Note: *$Cs_8[Nb_6O_{19}]\cdot 14H_2O$, T = 168 K.

**These values are averaged over all pairs within the Lindqvist ion structure.

References

1 Templeton, M. K. & Weinberg, W. H. Adsorption and Decomposition of Dimethyl Methylphosphonate on an Aluminum-Oxide Surface. *J. Am. Chem. Soc.* **107**, 97-108 (1985).

2 Taranenko, N., Alarie, J. P., Stokes, D. L. & Vo-Dinh, T. Surface-enhanced Raman detection of nerve agent simulant (DMMP and DIMP) vapor on electrochemically prepared silver oxide substrates. *J. Raman Spectrosc.* **27**, 379-384 (1996).

3 Halford, J. O., Anderson, L. C. & Kissin, G. H. The Raman spectra of the methyl alcohols, CH3OH, CH3OD, and CH2DOD. *J. Chem. Phys.* **5**, 927-932, doi:10.1063/1.1749965 (1937).

4 Hester, R. E. & Plane, R. A. RAMAN SPECTRA OF METHANOL SOLUTIONS .I. SATURATED SOLUTIONS OF SOME ELECTROLYTES. *Spectrochim. Acta Mol. Biomol. Spectrosc.* **A 23**, 2289-&, doi:10.1016/0584-8539(67)80121-1 (1967).

5 Mammone, J. F. & Sharma, S. K. Raman Spectra of Methanol and Ethanol at Pressure up to 100 kbar. *J. Phys. Chem.* **84**, 3130-3134 (1980).

6 Yu, Y. *et al.* Complete Raman Spectral Assignment of Methanol in the C-H Stretching Region. *J. Phys. Chem. A* **117**, 4377-4384, doi:10.1021/jp400886y (2013).

7 Vanderve.Bj & Herman, M. A. VIBRATIONAL ANALYSIS OF METHYLPHOSPHONIC ACID AND ITS ANIONS .1. VIBRATIONAL-SPECTRA. *J. Mol. Struct.* **15**, 225-236, doi:10.1016/0022-2860(73)85006-9 (1973).

8 Inscore, F., Gift, A., Maksymiuk, P. & Farquharson, S. in *Chemical and Biological Point Sensors for Homeland Defense Ii* Vol. 5585 *Proceedings of the Society of Photo-Optical Instrumentation Engineers (Spie)* (eds A. J. Sedlacek, S. D. Christesen, T. VoDinh, & R. J. Combs) 46-52 (2004).

9 Vanderve.Bj & Herman, M. A. VIBRATIONAL ANALYSIS OF METHYLPHOSPHONIC ACID AND ITS ANIONS .2. NORMAL COORDINATE ANALYSIS. *J. Mol. Struct.* **15**, 237-248, doi:10.1016/0022-2860(73)85007-0 (1973).

10 Farquharson, S., Maksymiuk, P., Ong, K. & Christesen, S. D. in *Vibrational Spectroscopy-Based Sensor Systems* Vol. 4577 *Proceedings of the Society of Photo-Optical Instrumentation Engineers (Spie)* (eds S. D. Christesen & A. J. Sedlacek) 166-173 (2002).

11 Nyman, M. *et al.* Solid-state structures and solution behavior of alkali salts of the [Nb6O19](8-) Lindqvist ion. *J. Cluster Sci.* **17**, 197-219 (2006).
